# The Impact of Short-Term Exposure to Air Pollution on the Exhaled Breath of Healthy Adults

**DOI:** 10.3390/s21072518

**Published:** 2021-04-04

**Authors:** Ariana Lammers, Anne H. Neerincx, Susanne J. H. Vijverberg, Cristina Longo, Nicole A. H. Janssen, A. John F. Boere, Paul Brinkman, Flemming R. Cassee, Anke H. Maitland van der Zee

**Affiliations:** 1 Amsterdam UMC, Department of Respiratory Medicine, University of Amsterdam, Meibergdreef 9, 1105 AZ Amsterdam, The Netherlands; a.lammers@amsterdamumc.nl (A.L.); a.vanstuyvenbergneerincx@amsterdamumc.nl (A.H.N.); s.j.vijverberg@amsterdamumc.nl (S.J.H.V.); c.longo@amsterdamumc.nl (C.L.); p.brinkman@amsterdamumc.nl (P.B.); 2 Centre for Sustainability, Environment and Health, National Institute for Public Health and the Environment (RIVM), Antonie van Leeuwenhoeklaan 9, 3721 MA Bilthoven, The Netherlands; nicole.janssen@rivm.nl (N.A.H.J.); john.boere@rivm.nl (A.J.F.B.); flemming.cassee@rivm.nl (F.R.C.); 3 Institute for Risk Assessment Sciences, Utrecht University, Yalelaan 2, 3584 CM Utrecht, The Netherlands

**Keywords:** electronic nose, exhaled breath, healthy adults, air pollution, ultrafine particles, exposure, aviation, road traffic

## Abstract

Environmental factors, such as air pollution, can affect the composition of exhaled breath, and should be well understood before biomarkers in exhaled breath can be used in clinical practice. Our objective was to investigate whether short-term exposures to air pollution can be detected in the exhaled breath profile of healthy adults. In this study, 20 healthy young adults were exposed 2–4 times to the ambient air near a major airport and two highways. Before and after each 5 h exposure, exhaled breath was analyzed using an electronic nose (eNose) consisting of seven different cross-reactive metal-oxide sensors. The discrimination between pre and post-exposure was investigated with multilevel partial least square discriminant analysis (PLSDA), followed by linear discriminant and receiver operating characteristic (ROC) analysis, for all data (71 visits), and for a training (51 visits) and validation set (20 visits). Using all eNose measurements and the training set, discrimination between pre and post-exposure resulted in an area under the ROC curve of 0.83 (95% CI = 0.76–0.89) and 0.84 (95% CI = 0.75–0.92), whereas it decreased to 0.66 (95% CI = 0.48–0.84) in the validation set. Short-term exposure to high levels of air pollution potentially influences the exhaled breath profiles of healthy adults, however, the effects may be minimal for regular daily exposures.

## 1. Introduction

Exhaled breath analysis is a topic of research which has gained increased attention in the past few years, especially in the field of respiratory diseases. Important aspects for this interest are the non-invasive nature of breath sampling, and the possibility of analyzing both local and systemic processes. Exhaled breath contains volatile organic compounds (VOCs) that either have an endogenous (e.g., metabolic processes) or exogenous (from ambient air) origin. The endogenous VOCs are the ones of interest regarding biomarker discovery for medical testing purposes, having already shown their potential for discrimination between several respiratory diseases and distinct disease phenotypes [1].

One of the steps towards future clinical implementation of exhaled breath testing is to better understand which exogenous factors could potentially influence the composition of exhaled breath and, thus, should be taken into account when examining exhaled breath. This holds especially true for electronic nose (eNose) technology, a rapid exhaled breath detection technique based on multiple cross-reactive sensors. Such sensors resemble the powerful mammalian olfactory system [2], in which each sensor can detect multiple VOCs and, vice versa, a VOC can interact with multiple sensors. Therefore, eNose technology does not allow for the detection of individual compounds, making it challenging to identify their source and impossible to delineate their metabolomic pathways. However, by using pattern recognition algorithms, this relatively cheap and easy-to-use technique, is a promising tool for point of care testing, if properly validated and standardized [3]. Regarding validation, knowledge about the possible influences of environmental exposures on the exhaled breath profile is an important aspect [4].

Ambient air is always in direct contact with our respiratory system, making it a part of our exhaled breath but also a continuous exposure, possibly influencing metabolic pathways. Components in the ambient air that can have possible adverse health effects are air pollutants, cigarette smoke, and viral and bacterial agents. With the abundance of road traffic and aviation, air pollution is of increasing concern regarding human health. Previous literature has shown that particulate matter has adverse health effects on the respiratory and cardiovascular system, in which fine and ultrafine particles (UFP) can induce pulmonary as well as systemic inflammation [5,6,7,8] and oxidative stress [9,10]. Such processes have been shown to be reflected in exhaled breath patterns of ventilated rats and patients with asthma or chronic obstructive pulmonary disease (COPD) [11,12,13]. Therefore, we hypothesize that exposure to air pollution could be detected in the exhaled breath profile.

As part of our study on the effects of short-term exposure to air pollution [14], we collected exhaled breath profiles by an eNose. Our objective was to investigate the effect of short-term exposures of 5 h to air pollution near a major airport and two highways, with a focus on UFP on the exhaled breath profile of healthy young adults. We did this by discriminating pre and post-exposure measurements based on the exhaled breath profile and by testing the associations between individual eNose sensor signals and the exposure measurements.

## 2. Materials and Methods

### 2.1. Study Design

In this prospective study, young healthy adults were exposed to ambient air near Amsterdam Airport Schiphol and two nearby highways (Amsterdam, the Netherlands), between April and October 2018 [14]. Participants were exposed for 5 h on minimally two and maximally four days, with at least two weeks between exposures. During the exposure, participants performed intermittent cycling at low intensity on an ergometer for 20 min/h and were seated in between. Extensive air monitoring was conducted, from which 5 h averages were calculated. Before and after each exposure, exhaled breath was measured using an eNose, at the Amsterdam UMC location AMC (Amsterdam, the Netherlands). A petrol-fuelled hybrid car, equipped with a high-efficiency particulate air (HEPA) filter, was used for transport between the exposure site and the hospital (15 km distance, a 15–20 min drive).

### 2.2. Study Population

Participants were young adults, non-smokers for at least 1 year (<5 pack years = number of packs of cigarettes smoked per day * number of years the person has smoked) and had normal lung function (predicted forced exhaled volume in 1 s (FEV_1_) > 80%). Subjects were excluded when they had any (history of chronic) pulmonary or cardiovascular disease, hay fever, or when they lived in the vicinity of highly polluted areas: <2 km from Schiphol Airport, <300 m from a highway, or on a busy road (>10,000 vehicles/day). Volunteers were screened for their medical history, as well as lung (spirometry and fractional exhaled nitric oxide (FeNO)) and heart function (electrocardiography (ECG), blood pressure (BP) and heart rate). More details about the inclusion and exclusion criteria, as well as the screening assessments, were published previously [14].

### 2.3. Exposures

All participants were exposed for 5 h to the ambient air near Schiphol Airport (northwest of two runways, ~300 m away), two highways (~500 m away) and Amsterdam (~10 km). Two to four participants were exposed on the same day, in a mobile exposure laboratory, through which the outside ambient air was circulated. We aimed to expose each participant to different UFP levels, sources (e.g., aviation and road traffic), and compositions between exposure days, by considering the meteorological conditions (mostly wind direction, as the location of the exposure site was fixed) when scheduling their visits.

#### 2.3.1. Exposure Parameters

The air inside the exposure chamber was monitored, from which 5 h averages were calculated for the following: particle number concentrations (PNC); particle mass concentrations (PM ≈ PM2.5 meaning particles mainly <2.5 µm); nitrogen oxides (NO_x_, NO_2_); black carbon (BC); and carbon monoxide (CO). The total PNC was measured by a condensation particle counter (CPC) with d_50_ = 4 nm. Furthermore, we monitored the temperature (temp) and relative humidity (RH) inside the mobile exposure laboratory. More details about the exposure (monitoring equipment) were published previously [14].

#### 2.3.2. PNC Sources

Next, we estimated the contribution of different sources to the PNC levels using a positive matrix factorization (PMF) source apportionment model, as extensively described by Pirhadi et al. [15]. In short, the source contribution model was based on the 5 h averages of total PNC (measured by CPC), PNC size fractions (measured by a scanning mobility particle sizer), BC, NOx and CO. The aviation source was labeled as “total aviation” and was also divided into “take-offs” and “landings”. Furthermore, two traffic sources were distinguished, “airport traffic” (e.g., passenger busses, baggage trucks) and “road traffic” (e.g., highways around the airport), but were also analyzed as one labeled “total traffic”.

### 2.4. Exhaled Breath Analysis

**Figure 1:**
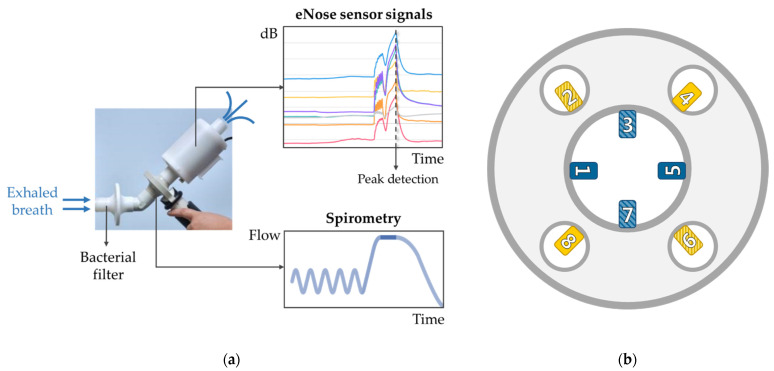
(**a**) The eNose measurement setup consisted of a mouthpiece, bacterial filter, spirometer and the SpiroNose. Participants rinsed their mouths with water three times and clipped their noses before performing 5 tidal breaths, a deep inhalation, a 5 s breath-hold, followed by a slow maximal exhalation (0.4 L/s). (**b**) Schematic overview of the SpiroNose (front view), with four sensor arrays on the outer side that detect the ambient air (**yellow**) and four on the inside that detect the exhaled breath (**blue**). All seven different sensor types are present fourfold, in which the arrays 1, 4, 5 and 8 (filled squares) contain sensors 1–4 (TGS2602, TGS2610, TGS2611-COO and TGS2600, respectively). Sensor arrays 2, 3, 6 and 7 (dashed squares) contain sensors 5–7 (TGS2603, TGS2620 and TGS2612, respectively).

Exhaled breath analysis was performed using an electronic nose (eNose), the SpiroNose, attached at the rear end of a spirometer (MasterScreen™ PFT, CareFusion) (Figure 1a). The SpiroNose consists of seven different cross-reactive metal oxide sensors: TGS2602 (sensor 1), TGS2610 (sensor 2), TGS2611-COO (sensor 3), TGS2600 (sensor 4), TGS2603 (sensor 5), TGS2620 (sensor 6), TGS2612 (sensor 7) (Figaro Engineering Inc., Osaka, Japan). Each sensor is present fourfold: twice on the inside, detecting the exhaled breath; and twice on the outer side of the device, detecting the ambient air (Figure 1b).

#### 2.4.1. eNose Measurements

First, subjects rinsed their mouth thoroughly three times with water. Subsequently, exhaled breath analysis was performed twice in a row, with a 2 min interval. The maneuver involved five tidal breaths, an inhalation up to total lung capacity, a breath-hold of 5 s and a slow (<0.4 L/s) maximal exhalation towards residual volume. During this maneuver, participants were breathing through a bacterial filter (Lemon Medical GmbH, Hammelburg, Germany) with their noses clipped. Furthermore, complaints of cough, dyspnea, sputum production and a blocked nose were assessed and were labeled as “no” when none and “yes” when one or more of these symptoms occurred.

#### 2.4.2. Data Processing

**Figure 2:**
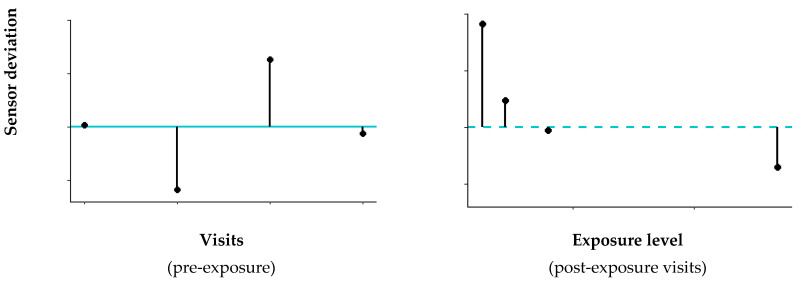
For the deviation percentage calculation, we first determined the individual mean sensor value based on all pre-exposure visits (blue solid line, **left graph**). Next, the deviation percentages from this mean were calculated for both the pre-exposure visits (left graph) and the post-exposure visits (**right graph**, with the blue dashed line representing the mean sensor value based on the pre-exposure visits).

The eNose signals were processed in MATLAB^®^ as described by De Vries et al. [16] and involved filtering, detrending, ambient air correction and peak detection. The highest sensor peak of the duplicate measurements was selected and normalized with respect to the most stable sensor, sensor 2, therefore data from sensor 2 are not presented. Further processing was performed in *R studio* and consisted of two steps in which we aimed to put the eNose sensor data in perspective with subject-specific pre-exposure baselines and fluctuations. First, the mean of all pre-exposure measurements (i.e., baseline) was calculated, after which the deviation from this baseline for all pre and post-exposure measurements, per subject and sensor were determined (Figure 2). The deviation is expressed as a percentage from baseline: (deviation/baseline)×100%. 

### 2.5. Statistical Analysis

All statistics were performed in R (version 3.6.1) and R studio (version 1.2.1335). The differences in exhaled breath profiles between pre and post-exposure were compared using discrimination analysis (using eNose sensor data) with R packages “mixOmics” and “pROC”. Associations between exposure levels and the change in eNose signal (using eNose deviation percentages) were analyzed with linear mixed-effects models using R package “lme4”. *p*-values < 0.05 were considered significant.

#### 2.5.1. Discriminant Analysis

**Figure 3:**
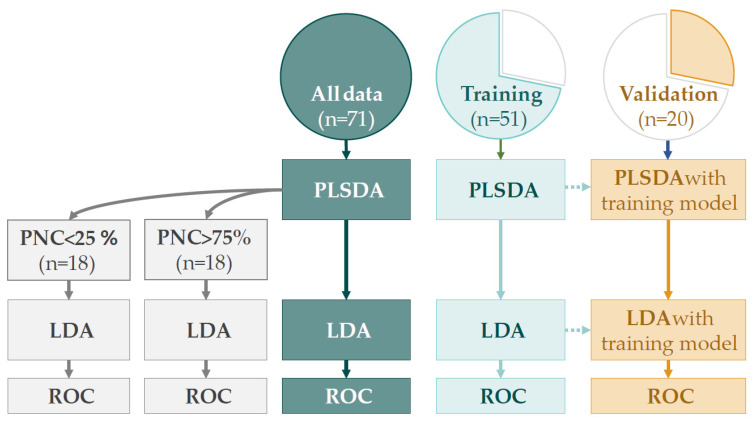
An overview of the discriminant analyses. Multilevel PLSDA and consecutive LDA models were constructed using all eNose sensor data and the training set, separately. The results from the PLSDA model, based on all eNose sensor data, was also used for the stratification analysis, in which high and low PNC exposures were compared. For the validation set, the same models as in the training set were used. The discrimination accuracy of all analyses was determined through ROC analysis. PLSDA = partial least square discriminant analysis; LDA = linear discriminant analysis; ROC = receiver operating characteristics; PNC = particle number concentration.

For the discriminate analysis, we performed multilevel partial least square discriminant analysis (PLSDA), in which the pre and post-exposure exhaled breath profiles are compared while taking into account the paired nature of the data (per visit, i.e., pre and post-exposure) to highlight the exposure effects within subjects. Next, the first two components of the PLSDA model were merged into one discriminant score using linear discriminant analysis (LDA). In addition, we stratified the results from the multilevel PLSDA into high (75% percentile, 18 visits) and low (25% percentile, 18 visits) PNC exposures, again followed by LDA, to test whether the discriminant performance was better for higher PNC exposure levels. Finally, we tested the robustness of the model by splitting the eNose sensor data in a training (≈70%) and validation set (≈30%). First, multilevel PLSDA and LDA models were constructed with the training set. Next, these obtained models were tested on the validation set. The performance of all discriminant analyses was determined by receiver operating characteristics (ROC) analysis, for which we reported the area under the ROC curve (AUROCC) with 95% confidence intervals (CI). An overview of the discriminant analysis is shown in Figure 3.

#### 2.5.2. Linear Mixed Effect Models

To take into account the paired data (pre and post), and the multiple visits per participant, we have constructed linear mixed effects models, per individual sensor. These models are suitable for the longitudinal analysis of within-individual change. For this analysis, we used eNose sensor data processed as deviations from a subject-specific mean baseline, expressed as percentages, as described before and depicted in Figure 2. 

The associations between the change in eNose deviation percentages and exposure were modelled using a linear mixed-effect model for each individual (*i*) and exposure day (*j*):

$$Y_{ij,\;post-pre}=\beta_{0}+\;Y_{ij,baseline}\;+\;\beta_{1}E_{j}+\;\beta_{2}V_{j}+U_{0i}+\varepsilon_{i}$$

The deviation percentage from the baseline for all post-exposure measurements (*Y_ij,post_*) was adjusted for the personal baseline (*Y_ij,baseline_*), as two participants with the same deviation but different baselines could have different deviation percentages. *E_j_* represents a vector of the exposure variable(s), *U*_0*i*_ the intercept for each participant (i.e., a random intercept) and *ε_i_* the error term. The *β*s represent population-average fixed effects, with *β*_0_ being the average change in the outcome parameters when all other covariates are zero, and *β*_1_ the average change in the outcome relative to a 5–95th percentile (5–95 p) increase in exposure. The vector *V_j_* represents the covariates that varied at each visit, which included the temperature and relative humidity in the exposure laboratory and the respiratory symptoms (i.e., cough, dyspnea, blocked nose or sputum production) that participants may have had before exposure (as a binary indicator “yes/no”).

We examined single-pollutant models for PNC, PM, BC, NO_2_ and CO. Next, we conducted two-pollutant models consisting of the PNC exposure adjusted for all other measured pollutants (BC, NO_2_, PM and CO) to investigate the independence of the effects associated with PNC [17]. Regarding the PNC sources, we investigated single-source models for take-offs, landings, airport traffic and road traffic, as well as total aviation (take-offs + landings) and total traffic (airport traffic + road traffic). Furthermore, we conducted a two-source model consisting of total aviation and total traffic. The fit of the models was examined by confirming a normal distribution of the residuals using Q–Q plots. Non-collinearity between covariates incorporated in the same model was verified using Pearson correlation (R < 0.6).

To facilitate the interpretation of the results from the linear mixed models, we determined the pre-exposure variability in the deviation percentages. For this, absolute deviation percentages of all pre-exposure measurements were averaged at an individual level and per sensor. Next, the median and interquartile range (IQR) of all participants was determined, again per sensor.

## 3. Results

### 3.1. Participants

Complete data were available for 20 out of the 23 participants of our main study. In accordance with our previous paper, we excluded two participants from the analysis because they received only one exposure [14]. In addition, one person was excluded as eNose data was missing for this person due to a sampling error. The participants were young adults (23 years, IQR: 20–23), mainly female (*n* = 16, 80%), with an average BMI of 22.7 kg/m^2^ (±2.4). They had normal lung function (FEV_1_ > 80% of predicted), FeNO levels (15, IQR: 12–23) and blood pressure (122 ± 12/77 ± 9 mmHg).

### 3.2. Exposures

In total, we conducted 32 exposure days, however, due to a sampling error, eNose data for 6 exposure days (i.e., 15 visits) were missing. We analysed 26 exposure days and a total of 71 visits, with four (*n* = 14; 70%), three (*n* = 3; 15%) or two (*n* = 3; 15%) visits per participant. The 5 h averages of all exposure variables are summarized in Table 1 and listed per day in Table S1. On average, the total PNC was 53,100 #/cm^3^ (range 12,600–173,200). At an individual level, the maximal contrast in PNC exposure that participants received (i.e., maximal–minimal exposure) was, on average, 77,600 #/cm^3^ (range 8800–152,600) (Table S2). The source apportionment model revealed that aviation, in particular aircraft take-offs, contributed more to PNCs (26,100 #/cm^3^; range 3200–101,800) than traffic-related sources (9100 #/cm^3^; range 1700–33,100) (Table 1). No multicollinearity existed between covariates included in the same model (R ≤ 0.58) (Table S3).

### 3.3. Discriminant Analysis

**Figure 4:**
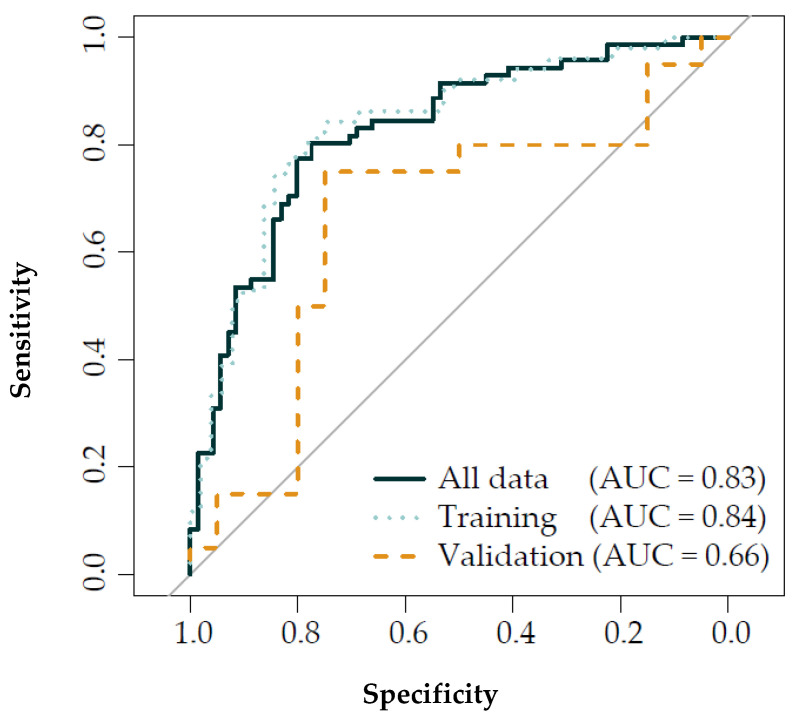
Receiver operating characteristic results from the multilevel PLSDA and LDA models. **Dark blue** solid = all data, 71 visits, **blue** dots = training set, 51 visits, and **orange** dashed line = validation set, 20 visits; PLSDA = partial least square discriminant analysis; LDA = linear discriminant analysis; AUC = area under the curve.

**Figure 5:**
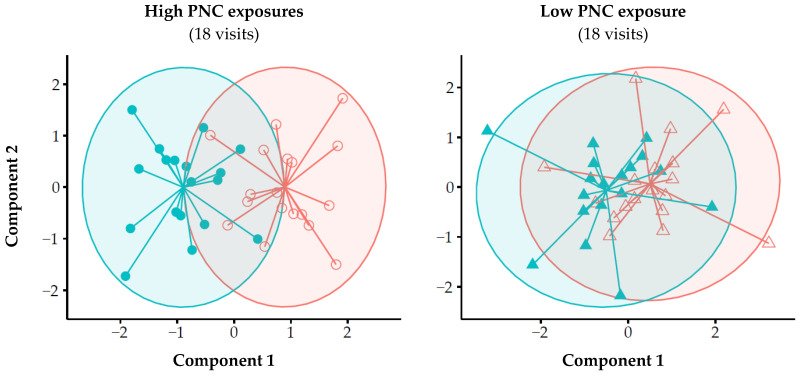
Results of the PLSDA model, stratified for high and low PNC levels, demonstrating that the difference in exhaled breath profiles between pre (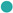 & 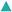) and post-exposure (○ & Δ) measurements were larger for high PNC exposures (**left**) compared to low PNC exposures (**right**). High levels were defined as PNC levels > 75% percentile (i.e., >71,900 #/cm^3^) and low < 25% percentile (i.e., <23,800 #/cm^3^). PLSDA = partial least square discriminant analysis; PNC = particle number concentration

The discrimination between pre and post-exposure using multilevel PLSDA combined with LDA reached an AUROCC of 0.83 (CI: 0.76–0.89). With the training set, a similar AUROCC of 0.84 (CI: 0.75–0.92) was reached, whereas the validation set reached an AUROCC of 0.66 (CI: 0.48–0.84) (Figure 4). Furthermore, stratification of the PLSDA components for the 25th and 75th percentiles in total PNC exposure (i.e., <23,800 and >71,900 #/cm^3^), showed to have a better accuracy for discriminating pre and post-exposure when PNC levels were high (AUROCC = 0.98, CI: 0.94–1.00) compared to low PNC levels (AUROCC = 0.77, CI: 0.61–0.93) (Figure 5, Table S4).

### 3.4. Linear Mixed Effect Models

#### 3.4.1. Pre-Exposure Variability eNose Deviations

The median pre-exposure variation in absolute deviation percentages differed between sensors, ranging between 3.9–13.2% with sensors 1, 5 and 7 having the highest variability; 6.8%, 6.4% and 13.2%, respectively (Table 2).

#### 3.4.2. Associations eNose Deviations and Pollutants

A significant inverse association was found between PNC and sensor 1 deviation percentages (−7.2% CI: −13.9–−0.5) (Table S5), which remained significant in all two-pollutant models (i.e., adjusted for PM, BC, NO_2_ and CO exposure) (Table S6). Furthermore, the deviation percentages of sensors 4 and 5 were significantly associated with PM exposure; (−5.7% CI: −10.8–−0.70) and (−12.1% CI: −23.5–−0.79), respectively. No significant association existed between deviation percentages and BC, NO_2_ and CO (Table S5).

#### 3.4.3. Associations eNose Deviations and PNC Sources

An inverse association was found between road traffic PNC source and sensor 6 deviation percentages (−5.0% CI: −8.2–−1.9) (Table S7). The total traffic PNC source was also inversely associated with the deviation percentages of sensor 6, both in the single-source (−5.3% CI: −8.9–−1.8) and two-source model (−5.3% CI: −8.8–−1.7) (Tables S8 and S9).

## 4. Discussion

Short-term exposures to air pollution, in particular UFP, near an airport and two highways, appeared to influence the exhaled breath profiles, detected by eNose technology, in young healthy adults. The discriminant analysis with all eNose sensor data and the training set resulted in good discrimination between pre and post-exposure, whereas the validation set reached a poor discriminant accuracy, possibly an indication of overfitting. The stratification analysis for PNC levels did demonstrate an exposure-response relationship, in which a larger difference in exhaled breath profiles existed between pre and post-exposure for higher PNC levels. Furthermore, we found robust associations between the deviation percentages of sensor 1 and total PNC, and between sensor 6 deviation percentages and the PNC sources “road traffic” and “total traffic”. However, the change in eNose deviations was in the range of pre-exposure variability. Altogether, air pollution could potentially be of importance as a confounder in exhaled breath analysis, depending on the exposure level and to which extent it affects the disease-related breath profile of patients with chronic airway diseases [4].

To our knowledge, there are no studies that published the effects of controlled exposures to air pollution on exhaled breath profiles detected by eNose technology. The study by Filipiak et al. did focus on (uncontrolled) exogenous factors influencing the composition of exhaled breath, such as smoking habits and exposure to air pollutants, using gas chromatography-mass spectrometry (GC-MS) analysis [18]. They collected breath from 46 healthy volunteers and 69 patients (both groups ≈50/50 smokers and non-smokers), in which the patients had lung cancer, either with or without COPD, or ear-nose-throat (ENT) cancer. The largest influence on exhaled breath was a smoking habit, however, also benzene, a component considered to originate from petrochemical industry products including gasoline, was detected in almost all breath samples. The small effect of air pollutants on the exhaled breath profile in our study can have several explanations. One of them could be that changes occurred in only one or a few VOCs that could not be detected by the eNose, but possibly could have been detected by GC-MS. Another explanation could be that the exposures did not trigger the production of VOCs associated with inflammation and oxidative stress, due to the short duration of the exposures and/or the very healthy and young population we have investigated.

The stratification for high and low PNC exposures in the discrimination analysis demonstrated a potential exposure-response relationship. However, our study involved extremely high PNC levels, up to 170,000 #/cm^3^, that are not representative of regular exposures to UFP. In highly urbanized areas or close to airports, PNC levels are commonly in the order of 10,000–20,000 #/cm^3^, with maximal PNC levels going up to 30,000–40,000 #/cm^3^ [19,20,21]. Therefore, air pollution may mainly be a possible confounder regarding exhaled breath analysis on days with extremely high levels of air pollution, due to e.g., massive use of fireworks [22,23,24] or smog. Regarding regular daily exposures to air pollution in urbanized areas, the effects on the exhaled breath profile may be minimal.

This study had several strengths. First of all, we performed multiple exposures in which we reached a large contrast in UFP levels within-subjects. This allowed for the investigation of exposure-response relationships within-subjects and the comparison of these to other participants. Secondly, we controlled air pollution exposures by making use of a mobile laboratory lab. This minimized the measurement error when compared to the commonly used central site monitoring. Thirdly, we used a source apportionment analysis to be able to distinguish UFP from different sources. Regarding the eNose sensor data, we examined normal deviations in exhaled breath profiles to put the effects of air pollution in perspective, as eNose sensor data is challenging to quantify. Finally, our statistical analysis takes into account the longitudinal character of our study, for the whole exhaled breath profile (i.e., discriminant analysis) and per sensor (linear mixed effect models).

Our study also had some limitations. We only compared single pre and post-exposure time points in which we may have captured only a snapshot of the response to the exposure. Furthermore, we had no information about the occupational and pre-visit exposure of the participants, which may have affected the pre-exposure measurement and the response to the exposure. However, we attempted to minimize this confounder by carefully selecting where our healthy volunteers lived; i.e., not near a highway, the airport or a busy street. In addition, the participants were mainly students with generally little occupational exposure. We, therefore, assume that the influence of occupational exposure on our results was minimal. Thirdly, our sample size was relatively small and based on the primary outcomes of the study (i.e., cardiopulmonary function). This possibly explains the issue of overfitting of the discriminant analysis models as demonstrated by the discrepancies between the discriminant accuracy of the training and validation set. Furthermore, our study mainly involved female participants, however, due to a lack of power we could not do a sensitivity analysis to investigate the influence of gender on our results. Finally, we may have faced the issue of multiple testing in our linear mixed-effect analysis, which has possibly led to finding significant associations by chance. Therefore, we chose to mainly focus on the consistency between the results from our different models [25].

For future research, it could be valuable to either use exhaled breath analysis in air pollution research, or to consider air pollution as a possible confounder in breath research. Exhaled breath analysis is a non-invasive and easy-to-perform method to detect both local and systemic metabolomic processes, making it an interesting tool in air pollution research. Regarding exhaled breath research, not focussing on the effects of air pollution, it could be important to take the air quality of a patient’s place of residence into account, mainly for patients living in highly polluted areas (e.g., cities facing recurrent smog). For this, one could make use of air quality data that are constantly monitored in many countries and are publicly accessible.

## 5. Conclusions

The short-term exposure of 5 h to air pollution, more specifically UFP, from air and road traffic may influence the exhaled breath profile, detected by eNose technology, of young healthy adults. Our study involved extremely high levels of UFP, as volunteers were exposed in high proximity to a major airport and two highways, which are not representative for regular daily exposures in urbanized areas. Therefore, more insight on air pollution as a possibly important confounder in breath analysis is required to determine to what extent it may influence the exhaled breath profile, especially with respect to the disease-related breath profile of patients with chronic airway diseases.

## Figures and Tables

**Table 1:** Exposure variables of all exposure days are based on 5 h averages.

	Exposure Days (*n* = 26)		
	Mean	5–95th Percentile	Range
**Pollutant**			
PNC (#/cm^3^)	53,100	19,300–138,200	12,600–173,200
PM (µg/m^3^)	24.6	14.9–41.3	13.6–47.5
BC (µg/m^3^)	0.59	0.21–1.26	0.13–1.55
NO_2_ (µg/m^3^)	27	13–44	12–48
CO (µg/m^3^)	634	516–782	494–830
**PNC sources**			
Total aviation (#/cm^3^)	26,100	3500–84,400	3200–101,800
Take-off (#/cm^3^)	16,600	700–58,800	500–62,000
Landing (#/cm^3^)	9500	1500–26,500	400–42,300
Total traffic (#/cm^3^)	9100	3600–16,700	1700–33,100
Airport traffic (#/cm^3^)	2400	500–4900	100–6800
Road traffic (#/cm^3^)	6700	2000–14,600	600–31,100
**Weather conditions**			
Temperature (°C)	24	20–27	19–29
Relative humidity (%)	54	43–66	40–66

PNC = particle number concentration; PM = particulate matter; BC = black carbon; NO_2_ = nitric oxide, CO = carbon monoxide; range = min–max. This table has partly been published previously for all exposure days (32 instead of 26) and without the PNC source information (Lammers et al., Environ Int 2020).

**Table 2:** Pre-exposure variability in absolute eNose deviation %.

Sensor	Deviation Percentage (%) Median (IQR)
1	6.8 (3.3–8.8)
3	3.9 (2.7–4.9)
4	4.3 (2.5–6.9)
5	6.4 (3.6–10.8)
6	4.7 (2.6–6.5)
7	13.2 (8.4–17.7)

At an individual level, the absolute deviation percentages of all pre-exposure measurements were averaged, per sensor. Next, the median, an IQR of all participants was determined, per sensor. IQR = interquartile range.

## Data Availability

The data presented in this study are available on request from the corresponding author. The data are not publicly available due to privacy restrictions.

## Supplementary Materials

The following are available online: https://www.mdpi.com/article/10.3390/s21072518/s1. Table S1. Distribution of exposure variables per exposure day (5 h averages); Table S2. Distribution of particle number concentrations per participant (5 h averages); Table S3: Correlation matrix. For all pollutants, particle size ranges and room conditions were measured during 5 h exposures; Table S4: Discrimination between pre and post-exposure; Table S5: Single-pollutant models. Associations between pollutants and eNose deviation percentages; Table S6: Two-pollutant models. Associations between PNC (corrected for other pollutants) and eNose deviation percentages; Table S7: Single-source models. Associations between PNC sources and eNose and deviation percentages; Table S8: Single-source models. Associations between PNC sources (totals) and eNose deviations; Table S9: Two-source model. Associations between adjusted PNC sources and eNose deviations.
