# Supplementary material for: The Impact of Short-Term Exposure to Air Pollution on the Exhaled Breath of Healthy Adults
Source: Sensors (Basel). 2021 Apr 4;21(7):2518. doi: 10.3390/s21072518 (PMC8038449; doi:10.3390/s21072518)
Supplement: Supplementary file 1 [file sensors-21-02518-s001.pdf]

Supplementary material

Title: The impact of short-term exposures to air pollution on the exhaled breath profile of healthy adults

Authors: A. Lammers, A.H. Neerincx, S.J.H. Vijverberg, C. Longo, N.A.H. Janssen, A.J.F. Boere, P. Brinkman, F. R. Cassee, A.H. Maitland – van der Zee

Table S1. Distribution of exposure variables per exposure day (5h averages)

Exposure day	PNC (#/cm³)	PM (μg/m³)	BC (μg/m³)	NO_2 (μg/m³)	CO (μg/m³)	Temp (°C)	RH (%)
1	74,466	29.9	0.3	12.4	619	19.4	43
2	40,156	17.0	0.3	13.3	599	18.8	44
3	28,898	18.8	0.6	48.4	775	22.3	54
4	22,049	27.3	0.8	37.8	650	23.9	62
5	23,356	22.4	0.7	28.1	621	24.2	51
6	18,861	39.4	1.3	41.4	784	22.3	62
7	27,835	14.5	0.5	24.5	619	21.9	46
8	30,413	13.6	0.3	16.4	587	21.4	43
9	134,879	26.6	0.7	42.8	569	24.0	40
10	32,144	25.7	0.5	12.8	510	24.0	53
11	23,474	25.7	0.2	16.1	691	24.0	50
12	35,357	28.0	0.4	27.3	667	25.2	54
13	24,866	16.0	0.3	16.3	494	24.6	50
14	12,619	21.9	0.4	20.0	579	24.1	58
15	20,926	27.9	0.1	12.4	537	25.2	57
16	52,896	16.0	0.3	23.3	611	26.0	52
17	39,531	18.2	0.3	27.0	568	26.5	47
18	38,360	18.4	0.4	18.8	557	25.0	47
19	46,866	47.5	0.8	26.8	616	26.6	53
20	45,524	20.3	1.0	27.7	603	28.6	52
21	64,379	27.8	0.5	24.8	670	24.8	65
22	139,321	24.4	1.0	36.2	705	25.2	66
23	173,187	19.1	0.6	33.9	597	21.3	58
24	128,166	24.8	1.1	35.8	681	24.4	66
25	80,856	26.7	0.6	34.1	744	26.6	63
26	20,644	42.0	1.6	44.0	830	22.8	62
**mean**	53,469	23.1	0.6	28.2	638	23.3	54
**SD**	43,776	8.3	0.4	12.2	83	2.7	7
**max**	173,187	47.5	1.9	60.2	830	28.6	66
**min**	10,520	10.6	0.1	12.4	494	15.7	40

Mass concentration based on filter measurements; PNC = particle number concentration; PM = particulate matter; BC = black carbon; NO_2 = nitric oxide; CO = carbon monoxide; DL= value below detection limit; Temp = temperature; RH = relative humidity; SD = standard deviation. This table has been published previously for all exposure days (32 instead of 26 days) (Lammers *et al.*, Environ Int 2020).

Table S2. Distribution of particle number concentrations per participant (5h averages)

Participant	PNC exposure (#/cm³)				Visits
	mean	min	max	contrast	
1	89,500	20,700	173,200	152,600	4
2	68,200	24,900	173,200	148,400	4
3	52,300	12,700	134,900	122,300	4
4	66,600	21,000	139,400	118,400	3
5	51,400	20,700	134,900	114,300	4
6	72,700	30,500	139,400	109,000	4
7	74,500	20,700	128,200	107,600	2
8	57,800	22,100	128,200	106,200	4
9	85,400	38,400	134,900	96,600	4
10	41,200	12,700	74,500	61,900	4
11	52,200	23,500	80,900	57,400	2
12	52,500	23,500	80,900	57,400	3
13	50,900	24,900	80,900	56,000	3
14	40,300	21,000	74,500	53,600	4
15	44,300	27,900	74,500	46,700	4
16	35,600	18,900	64,400	45,600	4
17	42,700	27,900	64,400	36,600	4
18	34,900	18,900	45,600	26,700	4
19	30,400	21,000	46,900	26,000	4
20	27,800	23,400	32,200	8,800	2
**mean**	**53,600**	**22,800**	**100,300**	**77,600**	
min	27,800	12,700	32,200	8,800	
max	89,500	38,400	173,200	152,600	

PNC = particle number concentration measured by condensation particle counter (CPC) with d_{50} = 4 nm. PNC levels are rounded to hundreds. Table is order based on the individual contrast in PNC exposure. This table has been published previously for all exposure days (32 instead of 26 days) (Lammers *et al.,* Environ Int 2020).

Table S3: Correlation matrix. For all pollutants, particle size ranges and room conditions measured during 5h exposures

	PNC[a]	PM	BC	NO_2	CO	Total aviation	Take-off	Landing	Total traffic	Airport traffic	Road traffic	Temp	RH
**PNC[a]**		-0.03	0.18	0.36	0.00	**0.97**	**0.96**	**0.83**	0.31	-0.18	0.35	0.01	0.09
**PM**	-0.03		0.59	0.34	0.48	-0.10	-0.05	-0.18	0.46	0.16	0.42	0.14	0.42
**BC**	0.18	0.59		**0.76**	0.67	0.11	0.19	-0.07	0.31	0.15	0.28	0.04	0.51
**NO_2**	0.36	0.34	**0.76**		0.67	0.33	0.40	0.12	0.21	0.32	0.14	0.15	0.40
**CO**	0.00	0.48	0.67	0.67		-0.02	-0.01	-0.03	-0.05	0.20	-0.10	-0.08	0.58
**Total aviation**	**0.97**	-0.10	0.11	0.33	-0.02		**0.97**	**0.87**	0.18	-0.14	0.21	0.05	0.08
Take-off	**0.96**	-0.05	0.19	0.40	-0.01	**0.97**		**0.74**	0.29	-0.01	0.30	0.20	0.12
Landing	**0.83**	-0.18	-0.07	0.12	-0.03	**0.87**	**0.74**		-0.09	-0.39	0.01	-0.26	-0.04
**Total traffic**	0.31	0.46	0.31	0.21	-0.05	0.18	0.29	-0.09		0.13	**0.97**	0.43	0.10
Airport traffic	-0.18	0.16	0.15	0.32	0.20	-0.14	-0.01	-0.39	0.13		-0.12	0.52	0.20
Road traffic	0.35	0.42	0.28	0.14	-0.10	0.21	0.30	0.01	**0.97**	-0.12		0.30	0.05
**Temp**	0.01	0.14	0.04	0.15	-0.08	0.05	0.20	-0.26	0.43	0.52	0.30		0.33
**RH**	0.09	0.42	0.51	0.40	0.58	0.08	0.12	-0.04	0.10	0.20	0.05	0.33	

Pearson correlations with in bold R >0.70; PNC = particle number concentration; PM = particulate matter; BC = black carbon; NO_2 = nitric oxide; CO = carbon monoxide; Temp = temperature; RH = relative humidity; a = PNC was detected by a condensation particle counter (CPC) with d_{50} = 4 nm; b = PNC size fractions were measured by a scanning mobility particle sizer (SMPS) with a limit of detection of 6-225 nm. This table has been partly published previously for all exposure days (32 instead of 26 days) and without the PNC source information (Lammers *et al.,* Environ Int 2020).

Discriminant analysis

Table S4: Discrimination between pre- and post-exposure

	**PLSDA multilevel + LDA**<br>AUROCC (95%CI)
**All eNose sensor data (n=71)**	**0.83** (0.76 – 0.89)
**PNC level stratification**	
PNC < 25 percentile (n=18)	0.77 (0.61 – 0.93)
PNC > 75 percentile (n=18)	**0.98** (0.94 – 1.00)
**Internal validation**	
Training set (n=51)	**0.84** (0.75 – 0.92)
Validation set (n=20)	0.66 (0.48 – 0.84)

In **bold** AUROCC > 0.80 and p-values < 0.05; PLSDA = partial least square discriminant analysis; LDA = linear discriminant analysis; AUROCC = area under the receiver operating characteristic curve; CI = confidence interval; eNose = electronic nose; n = number of visits; PNC = particle number concentration.

Pollutant models

Table S5: Single-pollutant models. Associations between pollutants and eNose deviation percentages

Sensor		PNC<br>5-95p = 118,900 #/cm³		PM<br>5-95p = 26.5 μg/m³		BC<br>5-95p = 1.05 μg/m³		NO_2<br>5-95p = 31 μg/m³		CO<br>5-95p = 265 μg/m³	
		Est. (95% CI)	R^2	Est. (95% CI)	R^2	Est. (95% CI)	R^2	Est. (95% CI)	R^2	Est. (95% CI)	R^2
Deviation %	**1**	**-7.2 (-13.9 – -0.5)**	0.12	5.6 (-2.5 – 13.79)	0.09	4.4 (-3.8 – 12.7)	0.08	1.1 (-7.0 – 9.1)	0.07	5.2 (-4.9 – 15.3)	0.08
	**3**	1.7 (-1.9 – 5.4)	0.16	-3.6 (-7.9 – 0.71)	0.18	-0.4 (-4.7 – 3.9)	0.15	-1.2 (-5.3 – 2.9)	0.15	2.3 (-3.0 – 7.6)	0.16
	**4**	1.3 (-3.1 – 5.7)	0.03	**-5.7 (-10.8 – -0.70)**	0.09	-1.1 (-6.1 – 4.1)	0.04	-2.3 (-7.2 – 2.6)	0.05	-2.1 (-8.5 – 4.2)	0.03
	**5**	1.5 (-8.3 – 11.3)	0.19	**-12.1 (-23.5 – -0.79)**	0.24	9.6 (-1.6 – 20.8)	0.22	4.5 (-6.5 – 15.4)	0.20	-3.6 (-17.9 – 10.7)	0.19
	**6**	-3.0 (-7.6 – 1.5)	0.04	-4.9 (-10.2 – 0.44)	0.06	0.5 (-4.8 – 5.9)	0.02	-0.2 (-5.4 – 4.9)	0.02	3.1 (-3.5 – 9.7)	0.03
	**7**	-1.0 (-19.0 – 17.0)	0.12	11.4 (-9.8 – 32.71)	0.14	17.1 (-3.4 – 37.5)	0.16	1.5 (-18.5 – 21.4)	0.12	0.7 (-25.2 – 26.6)	0.12

Data are presented as estimates (est.) with 95% confidence intervals (CI) intervals and the conditional explained variance (R^2) by both fixed and random factors (i.e. the entire model). All effect estimates are scaled to the 5-95th percentile change in the exposure of interest and are adjusted for respiratory symptoms, room temperature and room humidity. All results are adjusted for the individual baseline eNose signal (i.e. mean of all pre-measurement per subject and sensor). Numbers in **bold** are significant effects (p<0.05) and/or R^2>25%. *Exposures*: PNC = particle number concentration; PM = particulate matter; BC = black carbon; NO_2 = nitric oxide; CO = carbon monoxide.

Table S6: Two-pollutant models. Associations between PNC (corrected for other pollutants) and eNose deviation percentages

Sensor		PNC (5-95p = 118,900 #/cm³)							
		Adjusted for PM		Adjusted for BC		Adjusted for NO_2		Adjusted for CO	
		Est. (95% CI)	R^2	Est. (95% CI)	R^2	Est. (95% CI)	R^2	Est. (95% CI)	R^2
Deviation %	**1**	**-6.9 (-13.5 – -0.2)**	0.13	**-7.8 (-14.5 – -1.1)**	0.14	**-8.5 (-15.7 – -1.4)**	0.13	**-6.9 (-13.7 – -0.2)**	0.13
	**3**	1.5 (-2.1 – 5.1)	0.18	1.8 (-1.8 – 5.5)	0.16	2.4 (-1.4 – 6.3)	0.17	1.8 (-1.8 – 5.5)	0.16
	**4**	0.9 (-3.3 – 5.2)	0.09	1.5 (-2.9 – 5.9)	0.03	2.3 (-2.3 – 7.0)	0.04	1.2 (-3.2 – 5.6)	0.03
	**5**	0.7 (-8.9 – 10.2)	0.23	0.3 (-9.4 – 10.1)	0.22	0.1 (-10.3 – 10.6)	0.20	1.3 (-8.5 – 11.2)	0.19
	**6**	-3.4 (-7.8 – 1.0)	0.09	-3.1 (-7.7 – 1.4)	0.04	-3.4 (-8.2 – 1.5)	0.04	-2.9 (-7.4 – 1.6)	0.05
	**7**	-0.2 (-18.1 – 17.7)	0.14	-3.3 (-21.2 – 14.5)	0.15	-1.7 (-21.1 – 17.7)	0.12	-0.9 (-19.0 – 17.1)	0.12

Data are presented as estimates (est.) with 95% confidence intervals (CI) intervals and the conditional explained variance (R^2) by both fixed and random factors (i.e. the entire model). All effect estimates are scaled to the 5-95th percentile change in the exposure of interest and are adjusted for respiratory symptoms, room temperature and room humidity. The results of the deviation percentages were also adjusted for the individual baseline eNose signal (i.e. mean of all pre-measurement per subject and sensor). Numbers in **bold** are significant effects (p<0.05) and/or R^2>25%. *Exposures*: PNC = particle number concentration; PM = particulate matter; BC = black carbon; NO_2 = nitric oxide CO = carbon monoxide. PNC was detected by a condensation particle counter (CPC) with d_{50} = 4 nm.

PNC source models

Table S7: Single-source models. Associations between PNC sources and eNose deviation percentages

	Sensor	Take-off 5-95p = 58,100 #/cm^3		Landing 5-95p = 25,000 #/cm^3		Airport traffic 5-95p = 4,400 #/cm^3		Road traffic 5-95p = 12,600 #/cm^3	
		Est. (95% CI)	R^2	Est. (95% CI)	R^2	Est. (95% CI)	R^2	Est. (95% CI)	R^2
**Deviation %**	**1**	-6.7 (-14.1 – 0.7)	0.10	-5.0 (-11.7 – 1.7)	0.09	6.4 (-1.7 – 14.6)	0.10	-1.9 (-7.1 – 3.2)	0.07
	**3**	2.0 (-1.9 – 6.0)	0.16	3.1 (-0.4 – 6.7)	0.18	-0.6 (-4.9 – 3.8)	0.15	-1.4 (-4.1 – 1.3)	0.16
	**4**	2.3 (-2.5 – 7.0)	0.03	1.8 (-2.5 – 6.1)	0.04	-2.7 (-7.8 – 2.5)	0.06	-2.3 (-5.5 – 1.0)	0.08
	**5**	3.8 (-7.0 – 14.5)	0.20	0.9 (-8.8 – 10.5)	0.19	-3.4 (-15.2 – 8.3)	0.20	-4.9 (-12.2 – 2.4)	0.21
	**6**	-1.9 (-6.9 – 3.1)	0.03	-1.8 (-6.3 – 2.7)	0.03	3.1 (-2.2 – 8.4)	0.04	**-5.0 (-8.2 – -1.9)**	0.13
	**7**	4.4 (-15.1 – 24.0)	0.13	-6.9 (-24.7 – 10.8)	0.13	7.9 (-13.4 – 29.3)	0.13	-3.8 (-17.4 – 9.7)	0.13

Data are presented as estimates (est.) with 95% confidence intervals (CI) intervals and the conditional explained variance (R^2) by both fixed and random factors (i.e. the entire model). All effect estimates are scaled to the 5-95th percentile change in the exposure of interest and are adjusted for respiratory symptoms, room temperature and room humidity. The results of the deviation percentages were also adjusted for the individual baseline eNose signal (i.e. mean of all pre-measurement per subject and sensor). Numbers in **bold** are significant effects (p<0.05) and/or R^2>25%.

Table S8: Single-source models. Associations between PNC sources (totals) and eNose deviations

	Sensor	Total aviation 5-95p = 81,000 #/cm³		Total traffic 5-95p = 13,100 #/cm³	
		Est. (95% CI)	R^2	Est. (95% CI)	R^2
Deviation %	1	-6.4 (-13.7 – 0.8)	0.10	-1.2 (-6.9 – 4.5)	0.07
	3	2.5 (-1.4 – 6.4)	0.17	-1.7 (-4.7 – 1.3)	0.16
	4	2.2 (-2.5 – 6.9)	0.03	-3.1 (-6.6 – 0.5)	0.10
	5	2.9 (-7.6 – 13.4)	0.19	-6.2 (-14.1 – 1.8)	0.22
	6	-1.9 (-6.8 – 2.9)	0.03	**-5.3 (-8.9 – -1.8)**	0.13
	7	0.6 (-18.6 – 19.9)	0.12	-3.1 (-17.9 – 11.7)	0.13

Data are presented as estimates (est.) with 95% confidence intervals (CI) intervals and the conditional explained variance (R^2) by both fixed and random factors (i.e. the entire model). All effect estimates are scaled to the 5-95th percentile change in the exposure of interest and are adjusted for respiratory symptoms, room temperature and room humidity. The results of the deviation percentages were also adjusted for the individual baseline eNose signal (i.e. mean of all pre-measurement per subject and sensor). Numbers in **bold** are significant effects (p<0.05) and/or R^2>25%. Total aviation = take-off + landing; total traffic = airport traffic + road traffic.

Table S9: Two-source model. Associations between adjusted PNC sources and eNose deviations

	Sensor	Total aviation 5-95p = 81,000 #/cm³ Adjusted for total traffic	Total traffic 5-95p = 13,100 #/cm³ Adjusted for total aviation	
		Est. (95% CI)	Est. (95% CI)	R^2
Deviation %	1	-6.4 (-13.7 – 1.0)	-0.3 (-6.0 – 5.3)	0.10
	3	3.0 (-0.8 – 6.9)	-2.2 (-5.2 – 0.8)	0.19
	4	2.9 (-1.6 – 7.7)	-3.5 (-7.0 – 0.1)	0.10
	5	4.7 (-5.8 – 15.2)	-6.9 (-15.0 – 1.2)	0.22
	6	-0.6 (-5.4 – 4.1)	**-5.3 (-8.8 – -1.7)**	0.13
	7	1.3 (-18.2 – 20.9)	-3.3 (-18.3 – 11.7)	0.13

Data are presented as estimates (est.) with 95% confidence intervals (CI) intervals and the conditional explained variance (R^2) by both fixed and random factors (i.e. the entire model). effect estimates are scaled to the 5-95th percentile change in the exposure of interest and were adjusted for respiratory symptoms, room temperature and room humidity. The results of the deviation percentages were also adjusted for the individual baseline eNose signal (i.e. mean of all pre-measurement per subject and sensor). Numbers in **bold** are significant effects (p<0.05) and/or R^2>25%. Total aviation = take-off + landing; total traffic = airport traffic + road traffic.
